# Extrinsic Subclavian Vein Compression after Osteosynthesis of a Midshaft Clavicular Fracture in an Athlete

**DOI:** 10.1155/2015/981293

**Published:** 2015-04-01

**Authors:** Luciano A. Rossi, Nicolas S. Piuzzi, Santiago L. Bongiovanni, Ignacio Tanoira, Gaston Maignon, Maximiliano Ranalletta

**Affiliations:** Institute of Orthopedics “Carlos E. Ottolenghi”, Italian Hospital of Buenos Aires, Potosi 4247, 1199 Buenos Aires, Argentina

## Abstract

Clavicle fractures are common injuries. Traditionally, nonsurgical management has been favored; however, recent evidence has emerged indicating that operative fixation produces lower nonunion rates, better functional outcomes, improved cosmesis, and greater patient satisfaction. Although clavicle fixation has been considered a safe procedure, several complications related to plate fixation have been reported. We report a case of a 21-year-old basketball player that had a vascular complication associated with internal fixation of a clavicle fracture. An external compression of the subclavian vein was attributed to a long screw of a precontoured clavicular plate. Although vascular complications associated with clavicle fixation are rare, they may be limb and even life threating. It is advisable that surgeons take measures to avoid them especially when placing the medial screws.

## 1. Introduction

Clavicle fractures are common injuries and account for approximately 2.6% to 5% of all fractures in adults [1, 2]. Middle-third fractures are the most common, representing about 80% of all fractures [1].

The most common mechanism for a clavicle fracture is a fall onto the ipsilateral shoulder, making athletes particularly prone to this injury [3]. Court-Brown et al. showed that clavicle fractures have the highest prevalence among sport related fractures [4].

Traditionally, nonsurgical management has been favored [5, 6]. However, recent evidence has emerged indicating that operative fixation leads to lower nonunion rates, better functional outcomes, improved cosmesis, and greater patient satisfaction compared with nonoperative treatment [7–10].

Among the complications related to the use of plate fixation, the most frequently reported are infection, plate failure, hypertrophic or dysesthetic scars, nonunion, and refracture after plate removal [11, 12]. Despite the close proximity of the neurovascular structures to the clavicle, vascular injuries after plate fixation are rarely reported [11, 12].

Subclavian external compression by a screw has never been reported to our knowledge. We report a case of a basketball player who developed an extrinsic compression of the subclavian vein by a screw after plate fixation of a midshaft fracture of the clavicle.

## 2. Case Report

**Figure 1:**
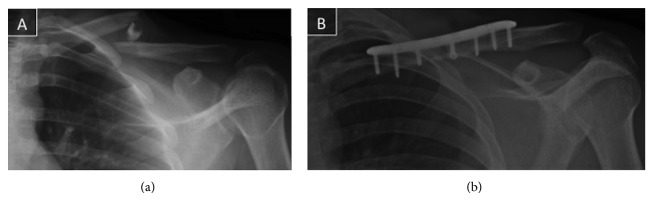
21-year-old male patient with basketball trauma. (a) He presents a type-2B-1 fracture. (b) Immediate postoperative radiographs. Reduction and osteosynthesis with an 8-hole precontoured locking plate using 6 screws, 3 lateral and 3 medial to focus.

A 21-year-old basketball player sustained a left fracture during a match. The radiographs exposed a Robinson's classification type-2B-1, and surgical treatment was performed 2 days after injury (Figure 1).

The patient was operated on in a beach-chair position under general anesthesia. Open reduction and internal plate fixation were performed. After reducing the fracture fragments, fixation was achieved with an eight-hole locked precontoured plate (Acumed (Oregon, USA)). The plate was applied to the bone with two cortical screws, one at each side of the fracture line. Subsequently, four locking screws completed the synthesis (Figure 1).

Postoperative rehabilitation protocol consisted of arm sling during the first two postoperative weeks, with sling removal four times a day to do pendulum exercises, allowing active elbow flexion-extension as tolerated. It was not permitted during the first three weeks to elevate the surgical arm above 90 degrees in any plane, and during the first six weeks it was not permitted to lift heavy weight. After week eight, full shoulder active ROM in all planes was allowed, with increase in strength intensity and functional training for gradual return to activities.

Four months after surgery, the patient reported alternative swelling and left upper extremity pain while raising repetitively his arm. The symptoms disappeared at rest and he had no pain or any other complaint regarding the shoulder.

Clinical examination showed a slightly swollen arm. No clinical signs of infection were evidenced. Pulses were present and symmetric. Both Wright and Allen maneuvers did not cause radial pulse loss. No collateral veins were observed. Passive and active shoulder's range of motion were normal.

Suspecting a late onset vascular complication related to the surgery, the subclavian and axillary vessels were studied. Initially, Echo Doppler examination showed a dynamic subclavian vein occlusion when the arm was elevated. Subsequently, a dynamic angio-computed tomography scan revealed a subclavian vein external compression related to the second screw (counting from medial to lateral) while the patient had his arm raised (Figure 3). The vein was permeable with the arm at a resting position.

**Figure 2:**
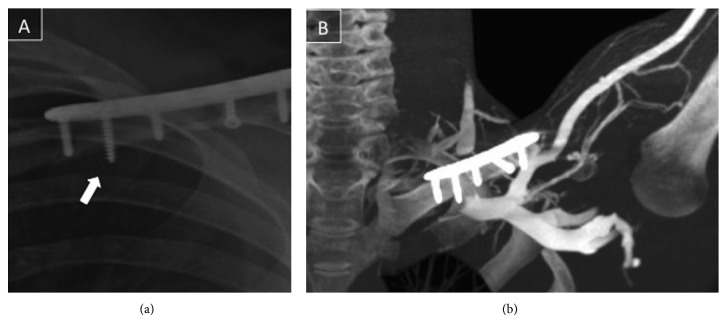
(a) Magnified image of the medial clavicle. Notice that the second screw from medial to lateral has 3 spirals protruding from the inferior face of the clavicle. See white arrow. (b) A dynamic angio-computed tomography scan revealed external compression of the subclavian vein in contact with the second screw from medial to lateral while the patient had his arm raised. See white arrow.

Although radiographic control showed a well-reduced fracture with hardware position unchanged (Figure 2), the second screw from medial to lateral had 3 spirals protruding from the inferior aspect of the clavicle.

**Figure 3:**
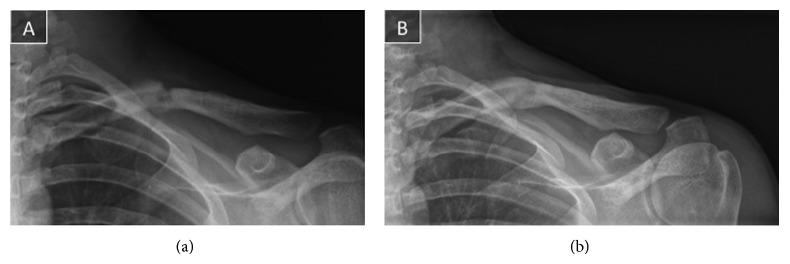
(a) Immediate postoperative radiograph after plate removal 5 months after the first surgery. (b) Six months after plate removal, the patient started contact sports. Complete radiographic consolidation is shown in the front clavicle radiograph.

Following the diagnosis of the external compression of the subclavian vein, the screws and the plate were removed (Figure 3).

After surgery, sling immobilization was indicated for a week and gradual return to daily activities was achieved. Contact sports were restricted for 6 weeks due to the potential risk of refracture. The patient returned to basketball practice at 6 months after the last surgery.

At last follow-up (one year after surgery), the patient had no arm swelling and remained free of symptoms. Echo Doppler examination revealed normal subclavian flux.

## 3. Discussion

Several recent prospective randomized clinical trials comparing nonoperative treatment with plate fixation for clavicle fractures showed that operative treatment improved functional outcomes and significantly decreased the incidence of long-term complications such as nonunion and symptomatic malunion [7–9, 13]. This has led to the increase in surgical management.

Though infrequent, neurovascular injuries are a recognized complication. They may be present as an acute or late complication, both in nonoperative and in operative scenarios [14–16]. Acute complication may be present after the transection of the vessel by the displaced fracture or as a late complication, secondary to compression from abundant callus formation [14–17].

The classic presentation is that of a young, athletic male presenting with acute onset of upper extremity pain and swelling in the dominant arm following a particularly strenuous activity [18, 19]. The majority of patients manifest with variable degrees of neck, shoulder, or axillary discomfort, arm heaviness, and pain associated with complaints of upper extremity swelling [20]. Swelling and pain typically improve with rest and elevation of the arm to the level of the heart, whereas elevation of the extremity overhead may aggravate the symptoms [21].

Coexistent signs related to brachial plexus compression (i.e., neurogenic thoracic outlet syndrome) may be present, manifesting as paraesthesias or pain in the ulnar nerve distribution, tenderness over the supraclavicular fossa, and wasting of the intrinsic hand muscles.

Clinical suspicion should be confirmed with imaging. Venography provides the best definition of abnormal venous anatomy and is the standard with which other modalities are compared [22, 23]. However, a recent systematic review showed that ultrasonography is an acceptable alternative to standard contrast venography [22]. Noncompressibility of the vein with or without visible intraluminal thrombus is the major criterion for the diagnosis of venous thrombosis. Duplex ultrasound is frequently used as the initial test for diagnosing upper extremity venous outflow obstruction because it is noninvasive and inexpensive and has an acceptable sensitivity and specificity for the diagnosis of upper extremity deep vein thrombosis [22, 24]. Less invasive methods of venography include computed tomographic (CT) and magnetic resonance (MR) venography [24, 25]. These modalities are not typically used to establish a diagnosis of upper extremity venous outflow obstruction. Rather, these studies are more useful for identifying anatomic abnormalities and causes of extrinsic compression such as tumors.

The treatment for upper and lower deep venous thrombosis is quite similar [26]. The treatment basis is anticoagulant agents having favorable reported results [27].

While attempting surgical treatment in a clavicle fracture, it is essential to consider that the clavicle has important adjacent anatomical structures, such as the subclavian vein and artery, brachial plexus, and pleura. There are different measures reported in the literature that reduce the risk of injury of the former mentioned anatomical structures at the time of surgery. These include the use of a blunt retractor placed under the clavicle during the drilling procedure, to place the plates anteriorly and to drill from anterior to posterior [28, 29]. Recently, the incorporation of locking plates allows unicortical fixation, which can also be considered as preventive measure.

Moreover, unicortical fixation using precontoured plates and locking screws has a similar biomechanical profile compared to gold standard nonlocked bicortical screws in cyclic axial compression and axial load to failure [30].

## 4. Conclusion

Although vascular complications associated with clavicle fixation are rare, they may be limb and even life threating. It is advisable that surgeons take measures to avoid them especially when placing the medial screws. In a patient with clinical suspicion of a vascular complication, it is essential not to delay imaging studies to confirm the diagnosis and to start prompt treatment to avoid serious complications.
